# Reverse engineering directed gene regulatory networks from transcriptomics and proteomics data of biomining bacterial communities with approximate Bayesian computation and steady-state signalling simulations

**DOI:** 10.1186/s12859-019-3337-9

**Published:** 2020-01-21

**Authors:** Antoine Buetti-Dinh, Malte Herold, Stephan Christel, Mohamed El Hajjami, Francesco Delogu, Olga Ilie, Sören Bellenberg, Paul Wilmes, Ansgar Poetsch, Wolfgang Sand, Mario Vera, Igor V. Pivkin, Ran Friedman, Mark Dopson

**Affiliations:** 1 Institute of Computational Science, Faculty of Informatics, Università della Svizzera Italiana, Via Giuseppe Buffi 13, Lugano, CH-6900 Switzerland; 2 Swiss Institute of Bioinformatics, Quartier Sorge – Batiment Genopode, Lausanne, CH-1015 Switzerland; 3 Department of Chemistry and Biomedical Sciences, Linnæus University, Hus Vita, Kalmar, SE-391 82 Sweden; 4 Linnæus University Centre for Biomaterials Chemistry, Linnæus University, Hus Vita, Kalmar, SE-391 82 Sweden; 5 Centre for Ecology and Evolution in Microbial Model Systems, Linnæus University, Hus Vita, Kalmar, SE-391 82 Sweden; 6 Luxembourg Centre for Systems Biomedicine, University of Luxembourg, Belvaux, Luxembourg; 7 Plant Biochemistry, Ruhr University Bochum, Bochum, Germany; 8 Center for Marine and Molecular Biotechnology, QNLM, Qingdao, China; 9 Faculty of Chemistry, Biotechnology and Food Science, Norwegian University of Life Sciences, Oslo, Norway; 10 College of Marine Life Sciences, Ocean University of China, Qingdao, China; 11 Faculty of Chemistry, Essen, Germany; 12 College of Environmental Science and Engineering, Donghua University, Shanghai, People’s Republic of China; 13 Mining Academy and Technical University Freiberg, Freiberg, Germany; 14 Institute for Biological and Medical Engineering. Schools of Engineering, Medicine & Biological Sciences, Pontificia Universidad Católica de Chile, Santiago, Chile; 15 Department of Hydraulic & Environmental Engineering, Pontificia Universidad Católica de Chile, Santiago, Chile

**Keywords:** Biological signalling simulations, Gene regulatory networks, Approximate Bayesian computation, Machine learning, Biomining, Acidophiles, Multispecies bacterial community interactions

## Abstract

**Background:**

Network inference is an important aim of systems biology. It enables the transformation of OMICs datasets into biological knowledge. It consists of reverse engineering gene regulatory networks from OMICs data, such as RNAseq or mass spectrometry-based proteomics data, through computational methods. This approach allows to identify signalling pathways involved in specific biological functions. The ability to infer causality in gene regulatory networks, in addition to correlation, is crucial for several modelling approaches and allows targeted control in biotechnology applications.

**Methods:**

We performed simulations according to the approximate Bayesian computation method, where the core model consisted of a steady-state simulation algorithm used to study gene regulatory networks in systems for which a limited level of details is available. The simulations outcome was compared to experimentally measured transcriptomics and proteomics data through approximate Bayesian computation.

**Results:**

The structure of small gene regulatory networks responsible for the regulation of biological functions involved in biomining were inferred from multi OMICs data of mixed bacterial cultures. Several causal inter- and intraspecies interactions were inferred between genes coding for proteins involved in the biomining process, such as heavy metal transport, DNA damage, replication and repair, and membrane biogenesis. The method also provided indications for the role of several uncharacterized proteins by the inferred connection in their network context.

**Conclusions:**

The combination of fast algorithms with high-performance computing allowed the simulation of a multitude of gene regulatory networks and their comparison to experimentally measured OMICs data through approximate Bayesian computation, enabling the probabilistic inference of causality in gene regulatory networks of a multispecies bacterial system involved in biomining without need of single-cell or multiple perturbation experiments. This information can be used to influence biological functions and control specific processes in biotechnology applications.

## Background

### Biomining

“Biomining” is the industrial process of exploiting acidophilic microorganisms for the recovery of valuable metals from sulfide mineral ores such as chalcopyrite [1, 2]. The process is catalyzed by microbial oxidation of ferrous iron that provides ferric ions for the chemical oxidation of metal sulfides and establishes a cycle between the ferric iron oxidative attack and biological oxidation of ferrous ions. Sulfur-oxidizing acidophiles also contribute to the process of mineral degradation by producing sulfuric acid from inorganic sulfur compounds. Compared to conventional metal recovery operations, biomining is less harmful to the environment [3]. It is therefore important to further optimize this process.

### Bacterial communities involved in biomining

Acidophilic microbes have different capabilities to generate energy from the conversion of mineral components under moderately thermophilic temperatures and are employed in commercial heap biomining operations [1, 4, 5]. Cooperative bioleaching occurs when the metabolic products of acidophilic microbes are utilized by other species and can occur by cell-cell direct contact or long-distance chemical gradients [4, 6]. Multispecies microbial communities are currently employed in biomining operations [4, 5]. However, the molecular details of the interactions between microbial cells are poorly characterized.

Typical acidophile species utilized during “bioleaching”, a term for the biomining process used when the metal of interest is part of the mineral matrix, include *Acidithiobacillus caldus* that is an obligate chemolithoautotrophic sulfur oxidizer that thrives at pH 2.5 [7, 8]; *Leptospirillum ferriphilum*, a ferrous iron oxidizing autotroph that is often the dominant iron-oxidizer in biomining environments at extremely low pH (1.3-1.6) and high redox potential conditions [9]; and *Sulfobacillus thermosulfidooxidans* that is a mixotroph primarily oxidizing iron but is also capable of oxidizing sulfur compounds at higher pH conditions compared to other acidophiles [10, 11]. The interplay between species in mixed acidophile communities at least partly determines the biomining efficiency and is therefore important to understand and optimize. In particular, the identification of biomolecular components involved in the process, both within a single species (intraspecies interactions) and between species (interspecies interactions), allows to unravel key biochemical processes for controlling microbial communities and metal dissolution. However, detailed analysis of the molecular interactions responsible for cross-talk between biomining species has not been carried out.

### Network modelling: reverse engineering OMICs data into GRNs

Next-generation sequencing (NGS) enables massive parallel sequencing that generates high-throughput data, for example, of an organism’s genome or transcriptome. Similarly, proteomics enable the large-scale analysis of an organism’s proteome. These OMICs data (named after their respective disciplines, i.e., genomics, transcriptomics, or proteomics) allow to quantify biological molecules of an organism in a holistic and comprehensive way. However, it remains challenging to understand relevant biological information from the vast amount of data generated by OMICs technologies and this is typically achieved by the quantification of features through computational pipelines and results in data tables containing information on gene expression [12–14]. These data are required to be further processed for identifying the underlying molecular interactions, especially when biological processes are distributed over multiple interacting cellular components. Network analysis is a powerful approach that identifies statistically significant interactions and represents molecular components such as genes or proteins as network nodes, interconnected by network edges, or links. Several modelling methods for network reconstruction exist [12, 15–21] and the outcome is a gene regulatory network (GRN) that is a synthetic representation of biological processes. The GRN can then be used for network interrogation, i.e., to predict biological functions in relation to the state of its network components [12]. The ability to infer not only GRNs nodes’ connectivity but also causality, represented by arrows (directed links) in network diagrams, is fundamental for network interrogation via forward simulations. Causality informs on the effect, direct or mediated by intermediates, of one node onto another. It also determines if a node is upstream or downstream in the cascade of events following a perturbation [15]. Forward simulations based on directed network diagrams allow to quantitatively determine the state of GRNs, and its associated biological function, as well as to predict its behaviour following perturbations of the network nodes [22–28].

### Steady-state signalling simulations

Different methods exist to perform simulations of GRNs that require a differently detailed description of the signalling interaction between network components, from highly detailed methods based on mass-action kinetics [22, 23, 29–32] to qualitative Boolean models [33, 34].

A knowledge-based computational framework for simulating biological networks has been developed that uses the assumption of steady-state between network components [24, 25]. The method only requires information on the nodes connectivity to make quantitative predictions on the network state and sensitivity to perturbations [26–28]. Steady-state simulations are commonly used in systems biology to perform *forward* simulations of directed networks in order to predict the behaviour of a network and its associated biological functions. Typical applications involve simulation of cellular signalling in complex diseases to study the effect of genetic dysfunctions such as gene mutations [26–28, 33], knockout/knockdown [24, 25], or the (combined) effect of therapeutic inhibitors [22, 23, 26, 28, 31, 32].

The computational tools used for *forward* simulations can also be employed for *reverse* simulations, i.e., to perform reverse engineering of experimental data [35] via e.g., Monte Carlo or Bayesian methods, where different combinations of model parameters are tested in their ability to reproduce the data observed experimentally [36]. However, this has not been applied to environmental microbiology data that often lack a detailed description of the underlying molecular interactions. In this case, reverse engineering can be achieved using steady-state *forward* simulations at the core of an inference model because they allow to integrate data of limited description details with standard parametrization and yet to provide a semi-quantitative analysis. This is in contrast to detailed models (e.g., mass-action models) that would require unavailable experimental information such as microscopic kinetic constants of the biochemical reactions; and also to Boolean models that provide a qualitative analysis and consequently cannot be compared to OMICs data in order to reverse engineer them into network diagrams.

### Approximate Bayesian computation

Approximate Bayesian computation (ABC) applies Bayesian inference without requiring an analytic expression of a likelihood function (as, for example, in Markov Chain Monte Carlo (MCMC) methods), which is typically limiting in complex systems. Instead, ABC approximates the likelihood function by using a model to simulate data in-silico by sampling model parameter values from a prior distribution. Simulated data are compared to experimentally measured data, also called observed data, through the Bayesian theorem and summary statistics, which represent the data with the maximum amount of information in the simplest possible form [36]. Based on a rejection algorithm, simulated data that are within a distance similarity range to observed data (e.g., by Euclidean distance) are retained to calculate the posterior probability distribution. The posterior probability distribution provides an estimate of the model parameters that best represent the observed data. This method could be applied to undirected networks and used to infer network causality, i.e., estimating the direction of network links, and therefore, obtaining directed networks that can be used for several modelling approaches in order to perform forward simulations of specific biological functions relevant in biotechnology applications.

In this study, we performed transcriptomics and proteomics experiments to identify genes and proteins involved in the formation of multispecies bacterial community interactions involved in bioleaching. We further used a steady-state *forward* simulation framework that relies on Hill-type interactions between molecular components using a standard parametrization that does not require the measurement of dynamic quantities underlying biochemical interactions, and use it as the core algorithm in ABC in order to infer causality in the GRNs of the bioleaching bacterial community.

## Methods

### Experimental methods

#### Microbial species cultivation

Three bacterial acidophile species were used in this study, *L. ferriphilum* DSM 14647^T^, *S. thermosulfidooxidans* DSM 9293^T^, and *A. caldus* DSM 8584^T^. Prior to the bioleaching experiments, cells were maintained at 38 ^∘^C in three separate axenic continuous cultures, maintaining the cells in the exponential growth state until inoculation. The continuous culture vessels (1 L working volume) contained Mackintosh basal salt (MAC) medium [37] and electron donor added in the form of 100 mM ferrous sulfate (*L. ferriphilum*, pH 1.4) or 5 mM potassium tetrathionate (*S. thermosulfidooxidans*, pH 2.3 and *A. caldus*, pH 2.0) adjusted to the designated pH values by addition of sulfuric acid. The continuous culture vessels, all tubing and MAC medium were autoclaved while the ferrous sulfate and potassium tetrathionate were sterile filtered (0.2 *μ*m pore size, cellulose acetate filter, PALL). Chalcopyrite mineral concentrate was provided by Boliden AB (Sweden) and originated from the Aitik copper mine (N 67^∘^4’ 24”, E 20^∘^ 57’ 51”). Prior to the experiment, chalcopyrite was sterilized as described in reference [38].

Bioleaching experiments were also conducted and analyzed as previously described [38]. In brief, quadruplets of 100 mL MAC medium (adjusted to pH 1.8 by addition of sulfuric acid) were supplemented with 2% (wt/vol) chalcopyrite concentrate and inoculated with combinations of the three bacterial species (10^7^ cells per mL per species), obtained by centrifugation from the continuous cultures (12,500 x g, 20 min) followed by cell counting using a Neubauer improved counting chamber. Cultures were incubated at 38 ±2^∘^C under slow shaking (120 rpm). Bioleaching experiments were terminated 14 days after the first onset of microbial oxidation of ferrous iron as indicated by a redox potential >400 mV *vs.* Ag/AgCl, resulting in total incubation times ranging from 14 to 20 days, after which the RNA and proteins were extracted.

#### RNA and protein extraction

For biomolecular extractions, the flasks were left to settle for 5 min. 75 mL supernatant was then mixed with an equal volume of sterile, ice-cold MAC medium. The sample was centrifuged at 12,500 x g for 20 min at 4 ^∘^C. The resulting cell pellet was washed twice by resuspending in sterile, ice-cold MAC, and then flash frozen in liquid nitrogen. Cell pellets were used for biomolecular extractions according to a previously published method [39], skipping the metabolite extraction step. A total of 30 RNA samples were then shipped on dry ice to the Science for Life Laboratory (Stockholm, Sweden) for sequencing, while the precipitated protein fraction of 44 samples was analyzed by mass spectrometry (data are available from the Fairdomhub repository at 10.15490/fairdomhub.1.investigation.286.1).

#### RNA sequencing and transcript analysis

RNA sequencing and analysis of the resulting reads was performed analogously to reference [38]. In short, rRNA depletion and libraries were prepared with the Illumina TruSeq Stranded mRNA kit before reads with an average length of 126 bases were generated on an Illumina HiSeq 2500 instrument. Raw reads were filtered with Trimmomatic v0.32 [40] and aligned to a concatenation of the three reference genomes (*A. caldus* DSM8584: GCF_000175575.2; *S. thermosulfidooxidans* DSM 9293: GCF_900176145.1; *L. ferriphilum* DSM 14647: GCF_900198525.1) with Bowtie-2 v2.3.2 [41]. Reads mapping to protein coding sequences were then counted with the FeatureCounts program of the subread package v1.5.1 [42]. The resulting read counts were converted to transcripts per million (TPM) separately for each of the three reference genomes to reflect relative gene expression per organism. A similar approach was pursued for intersample comparisons where read counts were normalized per reference genome [43] with DESeq2 v1.16.1 [44] and compared accordingly to obtain log _2_-fold changes (Log_2_FC).

#### Proteomics and protein identification

Five different protein extracts from continuous and three from batch cultures were precipitated in acetone, then dried and dissolved by vortexing in 20 *μ*L of 6 M urea – 2 M thiourea. Cysteines were reduced by incubation with 1 *μ*L 1 M dithiothreitol for 30 min at room temperature, and then alkylated with 1 *μ*L 550 mM iodoacetamide for 20 min in the dark. Afterwards, proteins were digested with lysyl endopeptidase (Wako) at a protease/protein ratio of 1:100 at room temperature for 3 h. Urea was diluted to 2 M with 50 mM ammonium bicarbonate for further trypsin digestion (sequencing grade; Promega) at a protease/protein ratio of 1:100 at room temperature for 12 h. Peptides were loaded onto stop-and-go extraction (STAGE) tips for storage, eluted from the tips, and shortly after analyzed by mass spectrometry [45].

Mass spectrometry for continuous-culture samples was performed by using an EASY-nLC 1000 liquid chromatography (LC) system (Thermo Scientific) and a Q-Exactive HF mass spectrometer (Thermo Scientific), as previously reported [46]. Mass spectra were recorded with Xcalibur software 3.1.66.10 (Thermo Scientific). Mass spectrometry for mineral culture samples was performed by using a nanoACQUITY gradient ultraperformance liquid chromatography (UPLC) pump system (Waters, Milford, MA, USA) coupled to an LTQ Orbitrap Elite mass spectrometer (Thermo Fisher Scientific Inc., Waltham, MA, USA). An UPLC HSS T3 M-class column (1.8 *μ*m, 75 *μ*m by 150 mm; Waters, Milford, MA, USA) and an UPLC Symmetry C 18 trapping column (5 *μ*m, 180 *μ*m by 20 mm; Waters, Milford, MA, USA) were used in combination with a PicoTip emitter (SilicaTip, 10 *μ*m internal diameter [i.d.]; New Objective, Woburn, MA, USA) for LC. Peptide elution was performed by using a linear gradient with increasing concentrations of buffer B (0.1% formic acid in acetonitrile [ULC/MS grade]; Biosolve, Netherlands) from 1% to 95% over 166.5 min, followed by a linear gradient from 1% acetonitrile within 13.5 min (1% buffer B from 0 to 10 min, 5% buffer B from 10 to 161 min, 40% buffer B from 161 to 161.5 min, 85% buffer B from 161.5 to 166.5 min, 95% buffer B from 166.5 to 167.1 min, and 1% buffer B from 167.1 to 180 min) using a flow rate of 400 nL min ^−1^ and a spray voltage of 1.5 to 1.8 kV. 2% buffer B was used to re-equilibrate the column for 15 min. The analytical column oven was heated to 55 ^∘^C and the desolvation capillary to 275 ^∘^C. The LTQ Orbitrap Elite instrument was operated according to instrument method files of Xcalibur (Rev.2.1.0) in the positive-ion mode. Linear ion trap and Orbitrap instruments were operated in parallel such that during a full MS scan on the Orbitrap instrument (in the range of 150 to 2000 m/z at a resolution of 60,000), tandem MS (MS/MS) spectra of the ten most intense precursors were detected in the ion trap from the most intense to the least intense using a relative collision energy for rapid collision-induced dissociation (rCID) of 35%. Mass spectra were recorded using a dynamic exclusion threshold with a repeat count of 1 and a 45-s exclusion duration window, such that ions with single or unknown charge were discarded for MS/MS, and subsequently processed with Xcalibur software 2.2 SP1.48 (Thermo Scientific).

Proteins from both continuous and mineral cultures were identified with Andromeda [47] and quantified with the label-free protein quantifications (LFQ) algorithm [48] included in the MaxQuant version 1.5.3.175 [46]. The FASTA protein database for identification was taken from the three reference genomes (see above). Perseus (v1.5.8.5) [49] was used for filtering and comparing of the normalized LFQ intensities. Rows with fewer than two values in either mineral or continuous cultures conditions were removed. The two conditions were then compared with two-sample Welch’s *t* test.

### Data analysis

#### Inference of undirected networks from transcriptomics and proteomics data

Correlation analysis was applied to the normalized transcriptomics and proteomics datasets, after filtering for genes that were differentially regulated with an associated P-value ≤0.05, using the R function cor() and the Pearson method in order to identify the links between nodes of the network. Unthresholded TPM and LFQ were used instead of Log_2_FC values in order to also allow links to be inferred between nodes representing genes in different bacterial species (interspecies links), in addition to intraspecies links. Partial correlation using the cor2pcor() R function from the corpcor package [50] was further used to discriminate between direct and indirect links identified by correlation analysis according to a described procedure [12, 16]. A stringent Pearson correlation threshold of *R*≥0.99 was used for attributing a link between two nodes, for both correlation and partial correlation. A more loose threshold increased the size of an undirected network by introducing more intermediates between interacting nodes, but conserved the connections between them (see Additional file 1: Figure S1).

#### Inference of directed networks from transcriptomics and proteomics undirected networks

For each of the GRNs analyzed in this study, an undirected network was used as reference for the nodes’ connectivity to create a set of directed networks that exhaustively covered all possible link directions. The number of directed networks is 2^*L*^, where *L* is the number of links. A computer simulation of each directed network was performed and in-silico generated data were compared to transcriptomics and proteomics data obtained experimentally. Computer simulations were performed by sampling each directed network accounting for an exhaustive combination of perturbations in the nodes activities. Each network simulated under a perturbation scheme was represented as a vector of normalized sensitivity values for each node, used as ABC’s summary statistics, and compared by Euclidean distance to a vector of normalized, scale-free Log_2_FC values determined experimentally from transcriptomics and proteomics for the genes corresponding to the simulated network nodes.

Simulations were compared to different experimental datasets differing in the composition of the bacterial cultures. Experimental perturbations were caused by the presence of other bacterial strains (mixed cultures) with respect to cultures grown with individual species (axenic growth of either *L. ferriphilum* or *S. thermosulfidooxidans* cultivated alone). Mixed cultures were composed of (i) *L. ferriphilum* and *S. thermosulfidooxidans* and (ii) *L. ferriphilum*, *S. thermosulfidooxidans*, and *A. caldus*.

#### Steady-state computer simulations

**Fig. 1:**
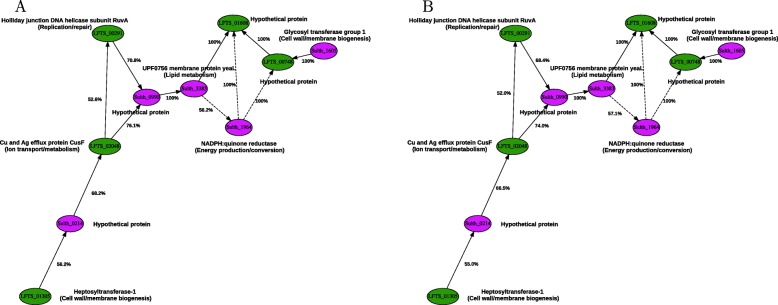
RNA cluster 1’s directed GRN estimated by ABC of computer simulations compared to different observed datasets. **a** Axenic cultures of *L. ferriphilum* or *S. thermosulfidooxidans* compared to their mixed culture, **b** axenic cultures of *L. ferriphilum* or *S. thermosulfidooxidans* compared to their mixed culture also containing *A. caldus*. Green and purple nodes represent genes belonging to *L. ferriphilum* and *S. thermosulfidooxidans*, respectively. Links with continuous (→) and dashed ($\dashrightarrow $) lines represent activation and inhibition interactions, respectively

**Fig. 2:**
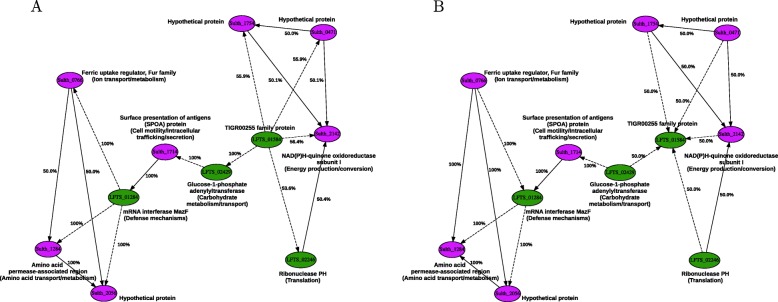
RNA cluster 2’s directed GRN estimated by ABC of computer simulations compared to different observed datasets. **a** Axenic cultures of *L. ferriphilum* or *S. thermosulfidooxidans* compared to their mixed culture, **b** axenic cultures of *L. ferriphilum* or *S. thermosulfidooxidans* compared to their mixed culture also containing *A. caldus*. Green and purple nodes represent genes belonging to *L. ferriphilum* and *S. thermosulfidooxidans*, respectively. Links with continuous (→) and dashed ($\dashrightarrow $) lines represent activation and inhibition interactions, respectively

**Fig. 3:**
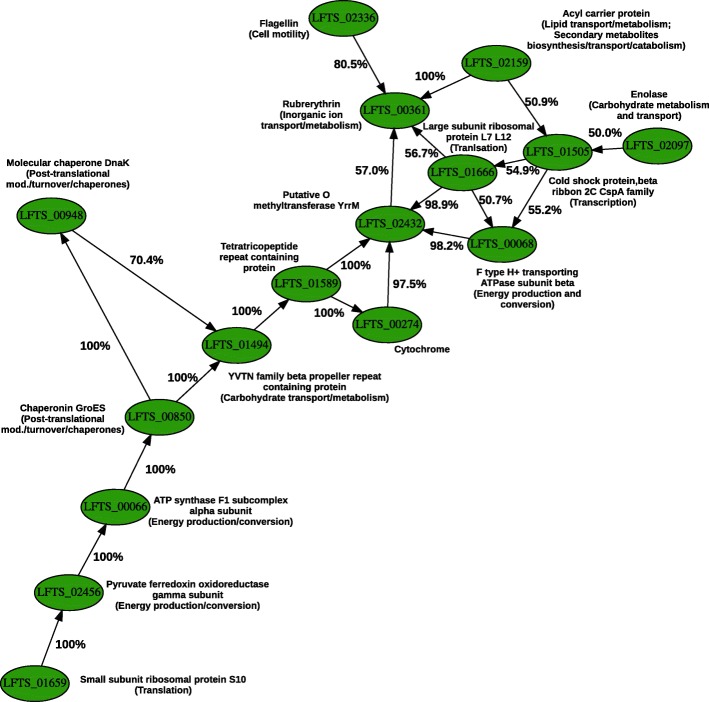
Protein cluster’s directed GRN estimated by ABC of computer simulations compared to the dataset obtained from axenic cellular cultures. Axenic cultures of *L. ferriphilum* compared to mixed cultures also containing *S. thermosulfidooxidans*. Links with continuous (→) lines represent activating interactions

The simulations used in this study rely on the computational method developed previously [24, 25] (the simulation program source code implemented in C++ and supported for Unix/Linux systems is available from the Fairdomhub repository at 10.15490/fairdomhub.1.investigation.286.1). GRNs were constructed as interaction diagrams composed of nodes and links. The nodes represent genes as a set of ordinary differential equations (ODEs) whose activity is modulated by the interaction of other genes in the network. Network links represent positive (Eq. 1) and negative (Eq. 2) interactions between the nodes, modelled according to an empirical Hill-type transfer function:

1 $$ Act(X \longrightarrow Y;\alpha,\gamma,\eta) = \alpha\frac{X^{\eta}}{X^{\eta}+\gamma^{\eta}}   $$

2 $$ Inh(X \dashrightarrow Y;\alpha,\gamma,\eta) = \alpha\frac{\gamma^{\eta}}{X^{\eta}+\gamma^{\eta}}   $$

where the Hill-exponent *η* is an empirical parameter widely used to quantify nonlinear signal processing [51–54]. Parameters *γ* and *α* determine a threshold of activation along the abscissa and a multiplicative scaling factor, respectively. Eq. 1 indicates the positive effect (activation) exerted by a source node *X* onto a target node *Y* (indicated by the arrow →), while negative interactions (inhibition) are represented by Eq. 2 (indicated by the arrow $\dashrightarrow $) as in Figs. 1, 2, and 3. The ODEs system that describes the GRNs evolves in time according to Eq. 3.

3 $$ \left\{\begin{array}{l} dX/dt = - \delta_{X}X + (\beta_{X} + \sum_{i} Act_{i}) \cdot \Pi_{j} Inh_{j} \\ dY/dt = - \delta_{Y}Y + (\beta_{Y} + \sum_{i} Act_{i}) \cdot \Pi_{j} Inh_{j} \\ \cdots \\ \end{array}\right.   $$

where every node (*X,Y*,...) in the model is parametrized by the parameters *β* and *δ* and every link by *α*,*γ*, and *η*. The parameter *β* accounts for the independent activity as a zero-order term added to each ODE, and *δ* for the decay of the biological species as a first-order decay term subtracted to the ODEs. When multiple links point to a single node, activation functions are added to each other while inhibition functions are multiplied by the current level of activity (see references [55, 56]). The simulation of a directed network yields the steady-state activity levels of the different nodes. The steady-state of the ODEs system corresponding to the simulated network was calculated numerically using the GSL library [57] (by use of *gsl_odeiv2_step_rk4*, which employs the explicit 4^*th*^ order Runge-Kutta algorithm), although this does not exclude that multiple steady-states might be present under certain parameter combinations.

The simulations used to reverse engineer the OMICs data were performed according to the coarse-grained method described in references [26–28]. For each directed network in the set derived from an undirected network, a simulation was performed that accounted for a perturbation in the activity of its nodes by increasing the nodes’ independent activity by a factor of 10 (*β*=0.01→0.1). This value is in the order of magnitude of the gene expression variation observed in our RNAseq and proteomics data, and roughly corresponds to the effect of genetic perturbations observed in other contexts [58, 59]. The other parameters of the model were set to unity.

This variation scheme was calculated for all combinations of the nodes in a directed network, in order to simulate the response to external perturbations, which may alter the activity of any combination of the genes. This was further applied to every directed network in the set derived from the undirected network of interest. In order to compare the in-silico generated data by the directed networks with the experimental dataset, sensitivity analysis was used as summary statistics for simulated data and compared to Log_2_FC values determined by transcriptomics or proteomics.

#### Sensitivity analysis used as summary statistics for ABC

In order to compare data generated in-silico with data obtained experimentally, sensitivity values were used as summary statistics in simulated data according to Eq. 4.

4 $$ {{\varepsilon}}^{Y}_{\phi} = \frac{\partial [ln(Y)]}{\partial [ln(\phi)]} = \frac{\phi}{Y} \cdot \frac{\partial Y}{\partial \phi} \approx \frac{\Delta [ln(Y)]}{\Delta [ln(\phi)]} = \frac{ln(Y_{i} / Y_{j})}{ln(\phi_{i} / \phi_{j})}  $$

where the sensitivity ${{\varepsilon }}^{Y}_{\phi }$ is represented as a function of the input parameter set *ϕ* and of the output variable *Y*. Eq. 4 expresses the relative change of activity in the nodes as a function of varying parameter sets. Two conditions (*i* and *j*) are evaluated at each step of the computational procedure according to the right-hand approximation. Applied to GRNs, the conditions were represented by vectors of steady-state values (*Y*_*i*_ and *Y*_*j*_) that correspond to the nodes’ activities and are determined by the parameter sets (*ϕ*_*i*_ and *ϕ*_*j*_).

Sensitivity values were calculated combinatorially over all possible network states, for each pair of conditions *i* and *j* that account for a change in node’s independent activity as described in Eq. 5.

5 $$ {{\varepsilon}}^{SS(N_{i})_{\beta(N_{j})=low} \: \rightarrow \: SS(N_{i})_{\beta(N_{j})=high} }_{{ \beta(N_{j})=low} \: \rightarrow \: \beta(N_{j})=high } = \frac{ ln \bigg \{ \frac{SS(N_{i})_{\beta(N_{j})=high} }{ SS(N_{i})_{\beta(N_{j})=low}} \bigg \} }{ ln \bigg \{ \frac{{\beta(N_{j})=high} }{{\beta(N_{j})=low}} \bigg \} }  $$

where *SS*(*N*) denotes the steady-state activity of a node *N* and *β*(*N*) its independent activity state. The arrow (→) indicates a change in condition.

A vector of sensitivity values (of size of the number of network nodes *n*) is calculated according to Eq. 5, scaled to a range of values ≤1, and compared by Euclidean distance to the vector of Log_2_FC measured experimentally and similarly scaled to values ≤1 [60].

### ABC sampling and rejection scheme

ABC is based on the Bayesian theorem of the conditional probability of model parameters (Θ) by knowing observed data (*D*) in relation to the probability of the data knowing the parameters. This is summarized in Eq. 6.

6 $$ P(\varTheta \mid D) = \frac {P(\varTheta \cap D)}{P(D)} = \frac{P(D \mid \varTheta)\cdot P(\varTheta)}{P(D)}  $$

where *P*(Θ∣*D*) is the posterior probability of the model parameters given the observed data, *P*(Θ) the prior probability for the parameters, *P*(*D*) the marginal likelihood (i.e., evidence, acting as a normalizing constant for *P*(Θ)), and *P*(*D*∣Θ) is the likelihood.

ABC is carried out by sampling from the prior distribution of model parameters, which allows the calculation of the posterior distribution through updates based on the observed data. Knowledge of model parameters can be included in the prior distribution, which represents the beliefs about the model parameters before the data are observed. If no information is available a priori for model parameters, a uniform prior distribution is used [36, 61]. Uniform prior distributions were chosen to define the directionality of the network links. Therefore, the causality of network links was fully determined by ABC based on the steady-state simulation model and no bias was introduced relying on previous knowledge of interacting genes. The rejection scheme used for calculating the posterior distribution of the link directionality was determined by thresholding the Euclidean distance between observed data and the data from simulated networks. A threshold was set such that only the top simulated data (<0.1*%* of the whole simulated dataset, ranked by Euclidean distance) best matching with observed data was considered. From the directed networks employed by the steady-state simulation model to generate the top simulated data, the proportion of links pointing in either directions was used to calculate the posterior distribution.

## Results and discussion

### Validation on single-cell literature data

To our knowledge, no single-cell data on bioleaching bacteria are currently available with such an accurate description of the underlying molecular interactions as in reference [15]. We therefore used this well-described molecular interaction system to test our causal link assignment method. The molecular system described in the PKC-PKA-MAPK-AKT signalling cascade has been studied extensively and therefore constitutes a solid ground truth for the validation of computational methods. Briefly, the data were collected by multicolor flow cytometry in order to observe multiple signalling proteins labelled with fluorescent antibodies. This allowed the simultaneous observation of the expression state of signalling pathway components in thousands of single cells. Single-cell data were further analyzed with a machine learning method based on Bayesian networks to elucidate the causal links between the measured signalling pathway components.

Our approach reverse engineered OMICs data from averaged cell populations (e.g., RNA transcript sequencing (RNAseq) and mass spectrometry protein data). We therefore condensed single-cell data of the published study data set corresponding to the experiments carried out without external perturbations (file “1.cd3cd28.csv”, consisting of 11 measured signalling proteins, and 852 observations) into average values for each of the measured signalling components, i.e., a vector of 11 elements corresponding to the signalling proteins, and challenged our computational method to infer correct link directionality from the undirected network of the published study. Our method successfully reproduced most of the published findings despite the information loss due to averaging single-cell data (see Table 1 compared with Fig. 3 A in article [15]).

**Table 1:** Comparison of the methodology applied to single-cell data [15] and our method on averaged data

Signalling	Posterior	Correctness	Agreement
interaction	probability (%)		with [15]
PLC → PIP2	25.5	n	n
PLC → PIP3	28.1	y	n
PIP3 → PIP2	58.7	y	y
PIP3 → AKT	35.4	n	y
ERK → AKT	70.8	y	y ^*^
PKC → JNK	100	y	y
PKC → P38	100	y	y
PKC → PKA	0	n	n ^*^
PKC → RAF	50.9	y	y
PKC → MEK	89.9	y	y
PKA → JNK	100	y	y
PKA → P38	100	y	y
PKA → RAF	100	y	y
PKA → MEK	100	y	y
PKA → ERK	100	y	y
PKA → AKT	100	y	y
RAF → MEK	48.3	n	n
MEK → ERK	87.5	y	y
PLC → PKC	95.6	y	y
PIP2 → PKC	57.3	y	n

Signalling interactions are represented by the molecular components of the signaling cascades detailed in reference [15]

*Inferred as novel in reference [15]

The posterior probability of a link pointing in the indicated direction in Table 1 is represented as a percentage fraction calculated from the top simulated data (<0.1*%* of the whole simulated dataset) best matching with the observed averaged data in reference [15]. A link pointing in a direction in 50% of the top directed networks indicates that our method was unable to discern the causality based on the data (the method predicts forward and reverse link direction with equal probability). The more the posterior probability deviates from 50% the more robust is the prediction of the link pointing in the indicated (>50*%*) or opposite (<50*%*) direction in Table 1.

For example, in agreement with the method of reference [15], the PKC–P38 interaction was strongly predicted by our method to point in the indicated direction (PKC → P38). In fact, the top <0.1*%* simulated data that best matches with observed data, was generated by simulating directed networks which all had that link pointing as PKC → P38. In other words, this single link set to point in the opposite direction, would be sufficient to cause disagreement between simulations and observed data. In contrast, the PKC → PKA link has a posterior probability evaluated at 0% indicating that our method strongly predicted the opposite directionality (PKC ← PKA), and is in disagreement with the published method [15]. Of note, the PKC–PKA link was identified as novel by the methodology presented in reference [15] and its inferred direction could not be clearly established [62]. More recent work also suggests complex interactions between PKA and PKC supporting a causality that depends on the different conditions the system is subject to [63]. These results prove that our proposed method was capable of assigning causality to undirected networks from averaged data with comparable accuracy as when employing an established method that use single-cell data. We therefore applied this method to our bioleaching OMICs data.

### Undirected network reconstruction from transcriptomics and proteomics data

In order to identify interspecies connections between genes involved in bioleaching, RNAseq gene transcript data of mixed cultures of *L. ferriphilum*, *S. thermosulfidooxidans*, and *A. caldus* were used to infer undirected GRNs (Additional file 1: Figure S2). The same procedure was applied to proteomics data to build undirected GRNs based on protein levels (Additional file 1: Figure S1). Two RNA (“*RNA cluster*” 1 and 2) and one proteomics (“*protein cluster*”) standalone undirected subnetworks of interest for biomining applications were selected based on their components involved in bioleaching. These sub-networks were composed of ≤16 nodes and ≤21 links, and were used to estimate the link causality by ABC (see the encircled undirected GRNs in Additional file 1: Figure S1A and S2).

### Reconstruction of directed networks from transcriptomics and proteomics undirected networks

RNA- and proteomics-based small undirected GRNs (RNA clusters 1 and 2 and the protein cluster) were used to generate an exhaustive set of directed networks with every possible link direction. Sampling this set by simulating each directed network allowed to select a subset of networks whose simulation outcome was close to the experimental data.

**RNA cluster 1** RNA cluster 1 was chosen from the set of undirected networks based on its computationally tractable size (10 nodes, 12 links), and the genes involved in bioleaching from both *L. ferriphilum* and *S. thermosulfidooxidans* that suggested potential interspecies cross-talk pathways. RNA cluster 1 comprised genes coding for transport of heavy metals in *L. ferriphilum* (e.g., LFTS_02048) plus *S. thermosulfidooxidans* genes involved in energy production (e.g., Sulth_1964). It also included genes involved in DNA repair and for membrane proteins that represent potential interest for the control of the bioleaching process.

The link directionality of RNA cluster 1 was estimated by ABC by comparing simulations to the experimental datasets. Link directionality was predicted to be the same independently of the experimental datasets used as a reference for ABC. However, the posterior probability for the network links was slightly different (Fig. 1). The accuracy of the methodology was evaluated using the Euclidean distance of simulated data to the observed ones, although it scales proportionally to the network size, making it difficult to compare the accuracy across different GRNs.

The simulations best matched the data obtained from the cultures of *L. ferriphilum* or *S. thermosulfidooxidans* cultivated alone compared to co-cultivation, i.e., the experimentally applied perturbation consisted of the presence of the other species in the culture (Fig. 1a). The Euclidean distance range of simulations to experimental data was [0.938912−2.46159] and the threshold for including a directed network in the posterior distribution set was a distance of 0.940989 that corresponded to a fraction of 0.0023% of the whole set derived from the undirected network model of RNA cluster 1 (488 simulated networks out of 20,971,520).

Similar results were obtained when simulated data were compared to axenic cultures of *L. ferriphilum* or *S. thermosulfidooxidans* with respect to their mixed cultures that also included *A. caldus* (Fig. 1b). Here, the simulation distance range was of [0.985189−2.36296] to experimental data, the threshold for including a directed network in the posterior distribution set was of 0.986991, corresponding to 0.0039% of the whole set derived from the undirected network model of RNA cluster 1 (812 simulated networks out of 20,971,520).

The similar prediction of link directionality and posterior probability estimated by ABC independent of the experimental datasets used as reference, supported the strength of the data and suggested that RNA cluster 1 represented an invariant set of gene interactions, constitutively active for bioleaching. While some causal links were predicted with a posterior probability estimate near 50%, indicating a weakly reliable estimate of a link direction based on the observed data (e.g., LFTS_01305 – Sulth_0214), others were estimated with stronger confidence (e.g., Sulth_3383 – LFTS_01608). In all cases, the heptosyltransferase-1 LFTS_01305 of *L. ferriphilum* involved in cell wall and membrane biogenesis was connected to the CusF copper and silver efflux protein LFTS_02048. This was potentially due to cell membrane changes required for metal efflux, via a *S. thermosulfidooxidans* hypothetical protein, therefore providing indications on uncharacterized or poorly annotated genes based on the inferred genes connectivity. Transcripts coding for the *L. ferriphilum* metal efflux protein (LFTS_02048) had a weak positive correlation on transcripts for the RuvA replication/repair protein LFTS_00291. This was likely due to copper inducing Fenton-like reactions that generate oxygen radicals that in turn cause DNA damage (reviewed in reference [64]). In addition, transcripts for the *S. thermosulfidooxidans* YeaL protein (Sulth_3383) involved in membrane lipid metabolism were also positively correlated to the CusF efflux protein potentially due to lipid peroxidation caused by the copper ions [65].

**RNA cluster 2** A second, larger cluster containing 11 nodes and 17 links was selected from the transcriptomics dataset based on similar criteria as for RNA cluster 1. Genes of potential relevance for multispecies bioleaching that were included in RNA cluster 2 comprised examples involved in energy production/conversion (Sulth_2142), in transport and trafficking (Sulth_1714, Sulth_1284, Sulth_0766), as well as in metabolic functions potentially involved in proton consuming reactions (LFTS_02429) and RNA interference mechanisms (LFTS_01284).

In general, the agreement between simulated data of RNA cluster 2 and the corresponding observed data appeared weaker compared to RNA cluster 1. Although the Euclidean distance scales with the network size, the overall Euclidean distance range between simulations of RNA cluster 1 and 2 compared to observed data of all experimental conditions was of [0.938912−2.36296] and [4.48968−6.84644], respectively.

Unlike RNA cluster 1, reconstruction of RNA cluster 2 showed a different link directionality depending on the experimental data it was compared to. Certain links were predicted to have an opposite causality depending on the experimental conditions. This was partly due that several links had a predicted posterior probability close to 50%. This indicated that based on the available data, the ABC method was incapable of reliably attributing a link direction. It also suggested that those genes interconnected by links with close to 50% predicted causality were part of complexes that are co-regulated in concert by a common factor, as supported by the dense interconnections that characterize the subclusters in the left and right side of RNA cluster 2. Interestingly, few genes that were predicted to have a different causality depending on different experimental conditions, were connected by links of a posterior probability higher than 50% (e.g., LFTS_01284 – Sulth_0766, Sulth_2056 – Sulth_1284, LFTS_01584 – LFTS_02429).

Simulations of RNA cluster 2 best matched experimental data from the axenic cellular cultures containing *L. ferriphilum* or *S. thermosulfidooxidans* alone when compared to their mixed cultures (Fig. 2a). The Euclidean distance range to experimental data was of [5.20429−6.45444] with an inclusion threshold for calculating the posterior distribution of 5.20431, corresponding to 0.00667% of the whole set derived from the undirected network model of RNA cluster 2 (6692 simulated networks out of 100,302,120).

The comparison of simulations to data of axenic cultures of *L. ferriphilum* or *S. thermosulfidooxidans* with respect to their mixed cultures that also included *A. caldus* was at a similar distance range of [5.53356−6.84644] (Fig. 2b). Here, a threshold of 5.53358 implied a set of best matching networks of 0.0163% used for computing the posterior probability of link causality (16,384 simulated networks out of 100,302,120).

The different link directions in RNA cluster 2 depended on the experimental data the simulations were compared to. This suggested a dynamic regulation of the GRN depending on the presence of *A. caldus* in the mixed culture. For instance, RNA transcripts coding for the *L. ferriphilummazF* mRNA interferase (LFTS_01284) strongly negatively correlated to the *S. thermosulfidooxidans* ferric uptake regulator (Fur; Sulth_0766) in axenic cultures of *L. ferriphilum* and *S. thermosulfidooxidans* compared to a mixed culture of the two species. In contrast, RNA transcripts for the *S. thermosulfidooxidans* Fur protein had a 100% negative correlation to *L. ferriphilum* MazF when the two species were in mixed culture also containing *A. caldus*. MazF is part of the MazEF suicide module involved in cell death due to e.g., DNA damage and oxidative stress [66]. The negative correlations between the Fur protein and a response to stress could be related to Fur being required when the ferric iron concentration was low and therefore, the stress response is not needed and *vice versa*. A second example of differently correlated RNA transcripts was for the *L. ferriphilum* TIGR00255 protein (LFTS_01584) that was positively or negatively correlated to RNA transcripts for several proteins dependent on the growth conditions or species present. However, the biological relevance of this correlation could not be discerned as the function of LFTS_01584 is unknown.

**Protein cluster** Due to a different efficiency between RNA and protein purification or to the fact that RNA transcript numbers do not always correlate to protein levels [67], it was not possible to find a network composed of the same differentially regulated genes and proteins in the respective datasets. Therefore, a protein cluster (16 nodes, 21 links) was chosen from the undirected network set reconstructed from the proteomics dataset. Differently from RNA clusters 1 and 2, it only included proteins involved in bioleaching from *L. ferriphilum*. This was due to the reduced number of proteins detected in the dataset and consequent limited number of GRNs derived in the undirected network set (Additional file 1: Figure S1). A single experimental dataset was available to be compared to simulated data. The protein cluster (Fig. 3) showed many *L. ferriphilum* genes interacting when axenic cultures of *L. ferriphilum* were compared to a mixed culture of *L. ferriphilum* and *S. thermosulfidooxidans*. The genes were coding for energy production (e.g., LFTS_00068), stress (e.g., LFTS_00850), translation (e.g., LFTS_01666), and cell attachment to the mineral (LFTS_02336) with positive correlations to each other.

The simulation distance range was of [9.60636−10.7846] with an inclusion threshold for posterior distribution calculation of 9.608 consisting of 0.0075%of the whole simulation set (82,781,763 simulated networks out of 1,099,511,627,776). The method could estimate causality of several network links with a posterior probability close to 100% indicating the relationships between proteins involved in bioleaching and intraspecies interactions of *L. ferriphilum* when grown in the presence of *S. thermosulfidooxidans*.

### Potential and limitations

Bayesian methods such as ABC with steady-state computer simulations at its core can be used in combination with correlations analysis to reverse engineer GRNs for which poor knowledge is available on the individual components. Steady-state models are well-suited because they require minimal information to set up a model. They only require information on the connections between the network nodes as for Boolean models, although they assume continuous regulation between the nodes [24, 27, 28]. Moreover, if experimental biological knowledge is available (e.g., kinetic parameters of interacting proteins), it can easily be integrated in the steady-state model [26] and the simulation procedure restricted to the relevant parameter ranges through the prior parameter distribution.

Importantly, the proposed approach only requires data generated from standard OMICs methods such as RNAseq and proteomics, as opposed to highly multi-dimensional data including multiple perturbations [17, 68, 69] or single-cell measurements [15, 70]. While the limited information contained in the datasets used in the present work typically allows only to reverse engineer undirected GRNs, ABC combined with steady-state model simulations allows to estimate causalities between network components and obtain directed GRNs.

The computational requirements of the presented method increase exponentially with the size of the processed network. This is due to the fact that, as a proof of principle in the current study, a set of directed networks was derived from an undirected network such that exhaustive sampling in the link directionality space was covered (2^*L*^, *L* being the number of links in the network), i.e., 20,971,520, 100,302,120 and 1,099,511,627,776 simulated networks for RNA clusters 1 and 2 and the protein cluster, respectively. However, this can be addressed by applying alternative random sampling schemes to explore large solution spaces when dealing with larger systems, such as Monte Carlo search [17], although the latter approach does not guarantee to find the optimal solution, as our exhaustive sampling did.

The proposed approach is limited to acyclic graphs, that constitute only a fraction of the total exhaustive space of possible directed networks [71]. This is a limitation of static Bayesian models that rely on the data used and the lack of information related to the variables evolving in time. In contrast, dynamic Bayesian models explicitly introduce time in experimental data and model interpretation, combined with the inclusion of perturbations (e.g., gene knockout), and allow to learn causal relationships between molecular components including feedbacks, although remaining unable to resolve *all* of the regulatory relationships [68, 69]. Moreover, the effect of noise, and irregular/undersampling is difficult to assess [72, 73].

ODE-based methods can be used as the core of machine learning methods such as MCMC, for which an analytic expression of a likelihood function is required, to infer topology and kinetic parameters from dynamic OMICs datasets [74]. However, this is sometimes limiting for complex systems and can be replaced by a sampling scheme using simulation models in ABC. On the other hand, simpler Boolean models can inform on the qualitative behaviour of potential networks that are underlying a specific biological function observed experimentally [33, 75, 76]. However, the simplicity of Boolean models might fail to capture complex regulatory effects.

The approach proposed in this work was able to infer link causality without requiring dynamic data. At the same time, the information required is comparable to the one used to set up Boolean networks. Moreover, static models such as Bayesian network structure learning, require a much larger number of observations than variables (*n*<<*p*, as in single-cell experiments) in order to estimate network causality [68, 69]. The strength of our method is that it allows to infer causality on a restricted data set of averaged values such as those typically obtained in OMICs experiments like RNAseq and proteomics. Although the undirected connectivity of the network remains to be determined with methods such as correlation analysis and can be inaccurate due to missing information measured [12, 16], the flux of the signal could be accurately determined with the presented method. In addition, the presence of intermediate components in the signalling network, that are not detected by OMICs experiments, does not affect the analysis dramatically as the steady-state simulation method is able to cope with missing information on non-detected intermediates. Although hidden confounders generally remain a potential problem in network reverse engineering, it was previously shown that consistent results could be obtained with an increase of 60% of the nodes in an analyzed network [25].

Therefore, the proposed method has the advantage of being conceptually simple, and the drawback to be highly computationally demanding. It is appropriate to studying a system that lacks an in-depth description of their molecular interactions. Unreliable gene annotation in GRNs can mislead the interpretation based on the causality estimated by the method. For example, in this study, Sulth_1714 was annotated as a surface antigen presentation protein in RNA cluster 2, which is unlikely to be correct in prokaryotic cells. This problem can be addressed by including additional information, considering that network structure determination can improve depending on the available information on the system with methods such as meta-analysis, data integration, etc. [12, 16].

## Conclusions

ABC combined with steady-state simulations was used to reverse engineer GRNs from OMICs data. The method required averaged data typically obtained in OMICs experiments such as RNAseq and proteomics. The approach was first validated on data of a published study. It was subsequently applied to RNAseq and proteomics data of mixed bioleaching bacterial cultures. Data could be reverse engineered into directed GRNs and causal relationships estimated probabilistically between genes of the same bacterial species (intraspecies interactions), as well as between species (interspecies interactions). This allowed to identify gene networks involved in bioleaching and the components that mediate multispecies bacterial community interactions. The method provides important means to identify unknown genes of poorly described systems and their role in the context of their network of interactions.

## Supplementary information

**Additional file 1** Supplementary Figures.

## Data Availability

Data and the simulation program source code implemented in C++ and supported for Unix/Linux systems is available from the Fairdomhub repository (10.15490/fairdomhub.1.investigation.286.1).
