# Supplementary material for: Reverse engineering directed gene regulatory networks from transcriptomics and proteomics data of biomining bacterial communities with approximate Bayesian computation and steady-state signalling simulations
Source: BMC Bioinformatics. 2020 Jan 21;21:23. doi: 10.1186/s12859-019-3337-9 (PMC6975020; doi:10.1186/s12859-019-3337-9)
Supplement: Supplementary file 1 — Additional file 1 Supplementary Figures. [file 12859_2019_3337_MOESM1_ESM.pdf]

SUPPLEMENTARY MATERIAL

Reverse Engineering Directed Gene Regulatory Networks from Transcriptomics and Proteomics Data of Biomining Bacterial Communities with Approximate Bayesian Computation and Steady-State Signalling Simulations

Antoine Buetti-Dinh[a,b,c,d,e], Malte Herold[f], Stephan Christel[e], Mohamed El Hajjami[g], Francesco Delogu[h], Olga Ilie[a,b], Sören Bellenberg[e], Paul Wilmes[f], Ansgar Poetsch[g,i], Wolfgang Sand[j,k,l], Mario Vera[m], Igor V. Pivkin[a,b], Ran Friedman[c,d], Mark Dopson[e]

[a]*Institute of Computational Science, Faculty of Informatics, Università della Svizzera italiana, Lugano, Switzerland*

[b]*Swiss Institute of Bioinformatics, Lausanne, Switzerland*

[c]*Department of Chemistry and Biomedical Sciences, Linnaeus University, Kalmar, Sweden*

[d]*Centre of Excellence for Biomaterials Chemistry, Linnaeus University, Kalmar, Sweden*

[e]*Centre for Ecology and Evolution in Microbial Model Systems, Linnaeus University, Kalmar, Sweden*

[f]*Luxembourg Centre for Systems Biomedicine, University of Luxembourg, Belvaux, Luxembourg*

[g]*Plant Biochemistry, Ruhr University Bochum, Bochum, Germany*

[h]*Faculty of Chemistry, Biotechnology and Food Science, Norwegian University of Life Sciences, Norway*

[i]*School of Biomedical and Healthcare Sciences, Plymouth University, Plymouth, United Kingdom*

[j]*Fakultät für Chemie, Biofilm Centre, University Duisburg-Essen, Essen, Germany*

[k]*College of Environmental Science and Engineering, Donghua University, Shanghai, People's Republic of China*

[l]*Mining Academy and Technical University Freiberg, Freiberg, Germany*

[m]*Institute for Biological and Medical Engineering. Schools of Engineering, Medicine & Biological Sciences. Department of Hydraulic & Environmental Engineering, Pontificia Universidad Católica de Chile, Santiago, Chile*

1. Supplementary Figures

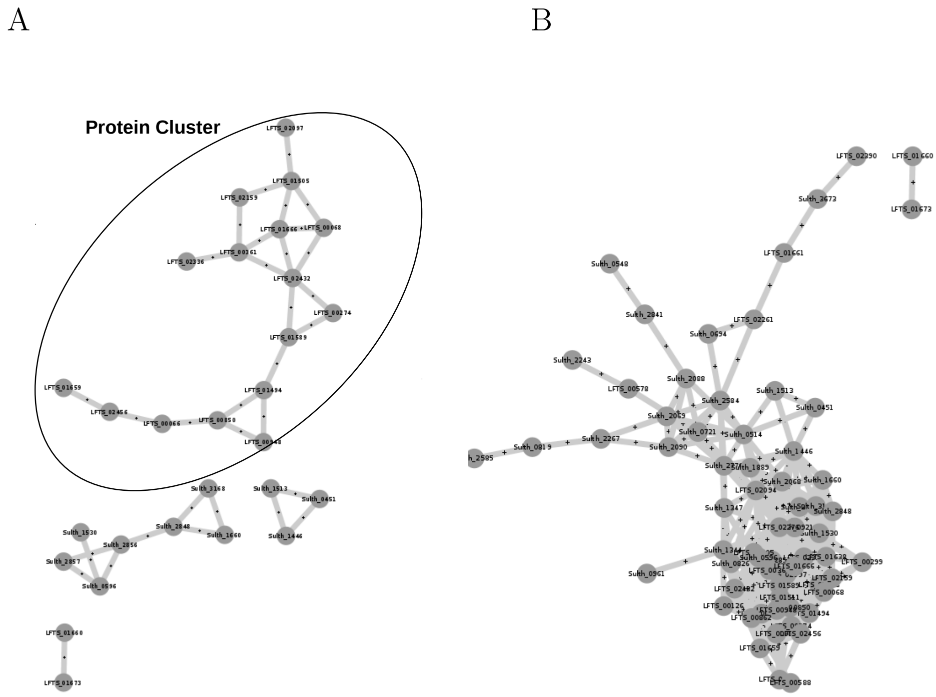

Figure S 1: Proteomics undirected network of the planktonic fraction of mixed cultures of *Leptospirillum ferriphilum* and *Sulfobacillus thermosulfidooxidans*. Threshold for correlation and partial correlation is: A) 0.99, network data are available in the figshare repository (DOI:10.6084/m9.figshare.7525436); B) 0.9, network data are available in the figshare repository (DOI:10.6084/m9.figshare.7525478). The cluster of connected nodes ("Protein cluster") selected for Approximate Bayesian computation (ABC) is encircled.

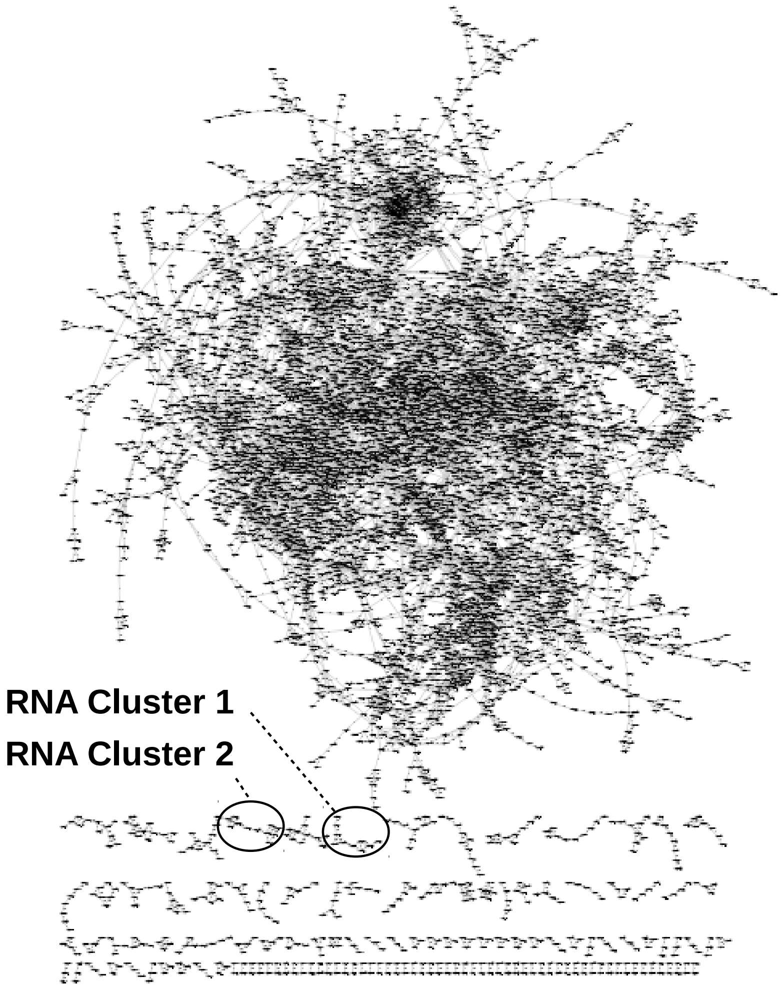

Figure S 2: Transcriptomics undirected network of mixed cultures of *Leptospirillum ferriphilum* and *Sulfobacillus thermosulfidooxidans* including both planktonic and sessile fractions. Threshold for correlation and partial correlation is 0.99. The network data are available in the figshare repository (DOI:10.6084/m9.figshare.7525430). The clusters of connected nodes ("RNA cluster 1" and "RNA cluster 2") selected for ABC are encircled.
